# Suppression of CB1 Cannabinoid Receptor by Lentivirus Mediated Small Interfering RNA Ameliorates Hepatic Fibrosis in Rats

**DOI:** 10.1371/journal.pone.0050850

**Published:** 2012-12-12

**Authors:** Si-Wen Chen, Ben-Yan Wu, Shi-Ping Xu, Ke-Xing Fan, Li Yan, Yuan Gong, Jun-Bao Wen, Dao-Hong Wu

**Affiliations:** 1 Department of Gastroenterology, NanLou Clinic, General Hospital of PLA, Beijing, China; 2 International Joint Cancer Institute, The Second Military Medical University, Shanghai, China; National Institutes of Health, United States of America

## Abstract

It is recognized that endogenous cannabinoids, which signal through CB1 receptors in hepatic stellate cells (HSCs), exert a profibrotic effect on chronic liver diseases. In this study, we suppressed CB1 expression by lentivirus mediated small interfering RNA (CB1-RNAi-LV) and investigated its effect on hepatic fibrosis in vitro and in vivo. Our results demonstrated that CB1-RNAi-LV significantly inhibited CB1 expression, and suppressed proliferation and extracellular matrix production in HSCs. Furthermore, CB1-RNAi-LV ameliorated dimethylnitrosamine induced hepatic fibrosis markedly, which was associated with the decreased expression of mesenchymal cell markers smooth muscle α-actin, vimentin and snail, and the increased expression of epithelial cell marker E-cadherin. The mechanism lies on the blockage of Smad signaling transduction induced by transforming growth factor β1 and its receptor TGF-β RII. Our study firstly provides the evidence that CB1-RNAi-LV might ameliorate hepatic fibrosis through the reversal of epithelial-to-mesenchymal transition (EMT), while the CB1 antagonists AM251 had no effect on epithelial-mesenchymal transitions of HSCs. This suggests that CB1 is implicated in hepatic fibrosis and selective suppression of CB1 by small interfering RNA may present a powerful tool for hepatic fibrosis treatment.

## Introduction

Hepatic fibrosis is a reversible wound-healing response characterized by an imbalance between excessive synthesis of extracellular matrix (ECM) and altered matrix degradation. The fibrogenic process is consecutive to proliferation and accumulation of myofibroblastic cells deriving from activated hepatic stellate cells (HSCs) and hepatic myofibroblasts (MFs). Both cell types express smooth muscle α-actin (α-SMA) and synthesize fibrogenic cytokines (transforming growth factor β1, TGF-β1), chemokines, fibrosis components (fibronectin, procollagen type I, and so on) and inhibitors of matrix degradation [1].

Endogenous cannabinoids are a family of molecules derived from arachidonic acid that signal through CB1 and CB2 receptors. Several studies have showed that chronic liver disease, including hepatic fibrosis, liver cirrhosis, alcoholic fatty liver and nonalcoholic fatty liver, all associated with the upregulation of endocannabinoids and their receptor, CB1 [2]–[10]. Increased activity of the hepatic CB1 also play a prominent role in both liver regeneration and liver carcinoma [11]. Major endogenous ligands of cannabinoids are anandamide, 2-arachidonylglycerol (2-AG), noladin ether and virodhamine [12]. It is recognized that endocannabinoids exert a profibrotic effect that is possibly mediated by CB1 receptors. This is compatible with the finding of increased CB1 expression in HSCs and hepatic MFs in the cirrhotic human liver and in the fibrotic livers of mice [13]. Genetic or pharmacological ablation of CB1 receptors protected mice against liver injury; this was reflected by the reduced expression of α-SMA and TGF-β1 [13]. The profibrotic effects of CB1 activation could provide a rationale for the use of CB1 antagonists in the medical management of advanced liver cirrhosis. And CB1 have increasingly emerged as crucial targets during liver diseases [13].

In this study, we inhibited the CB1 expression by RNA interference to block its intracellular signaling transduction and investigated its effect on the biological characteristics of HSCs in vitro, and aimed to examine the therapeutic effect of CB1 small interference RNA (siRNA) on chronic liver disease and consider their implications regarding disease mechanism and the development of new therapeutic modalities. Furthermore, we compared the effect of CB1 siRNA with CB1 antagonists on biological characteristics of HSCs in vitro, and present CB1 siRNA as a powerful tool for hepatic fibrosis treatment.

## Materials and Methods

### Lentivirus vectors for CB1 RNAi

Four different CB1-specific target sequences were chosen using the CB1 reference sequence (Gene Bank Accession No NM_012784). Double-stranded DNA were synthesized according to the structure of a pGCSIL-GFP viral vector (Genechemgene, Shanghai, China) and then inserted into a linearized vector. The positive clones were identified as lentiviral vectors named KD1, KD2, KD3 and KD4. Among the four vectors, KD4 (target sequence: 5′-GGAGACACAACAAACATTA-3′) induced the highest levels of downregulation. So KD4 vector and viral packaging system were cotransfected into 293 cells to replicate competent lentivirus. The lentivirus containing the rat CB1 shRNA (short hairpin RNA) expressing cassette was used as a positive control for lentivirus production and denoted as CB1-RNAi-LV in the next experiments. The pGCSIL/U6 mock vector was also packaged and used as a negative control, denoted as NC-LV, which has no significant homology to rat gene sequences. For Annexin V/PI detection, we modified the lentivirus with deleting the GFP tag. The titers averaged 1×10^8^ TU/mL.

### Cell culture and transfection

Primary HSCs were isolated from SD rats (about 400 g body weight) by in situ perfusion, followed by centrifugation on a discontinuous gradient of metrizamide, as described previously [14]. The isolated HSCs were identified by their intrinsic vitamin A autofluorescence and by staining for desmin. Their purity was >95%. Cells were seeded in Dulbecco's modified medium containing 10% fetal bovine serum. Activated HSCs were obtained by subcultivation of HSCs at day 7 and then the cells were plated on new culture dishes for screening the efficacy of CB1 shRNA.

To examine the effect of CB1-RNAi-LV on activation and ECM production of HSCs, primary HSCs were transduced with lentiviral vectors CB1-RNAi-LV or NC-LV after expansion. For transduction, cells were seeded at 2000 cells/cm^2^ in a T-75 cm^2^ flask. The following day virus particles were added at a multiplicity of infection (m.o.i.) of 40 for 36 hours. Then cells were washed and cultured continually. 60 hours later, freshmedium with CB1 antagonists AM251 (1 µM, Santa Cruz, Santa Cruz, USA) was added to the cultured cells. The following day fresh medium with the endogenous cannabinoid ligands 2-AG (1 µM, Tocris Bioscience, Bristol, UK) was added to the cultured cells. 24 hours later, cells were collected for reverse transcription-polymerase chain reaction (RT-PCR) and western blot analysis.

To explore the effect of CB1-RNAi-LV on proliferation and the expression of PDGF receptor β subunit (PDGFR-β) of HSCs, primary HSCs treated with 20 ng/ml platelet-derived growth factor-BB (PDGF-BB; Sigma, St Louis, USA) for 48 h were transduced with lentiviral vectors CB1-RNAi-LV or NC-LV for another 48 h. Cells were then collected and total RNA was isolated with Trizol reagent (Invitrogen, Carlsbad, USA).

### RT-PCR assay

cDNA was amplified from RNA with the use of the RT–PCR (Reverse transcriptional-PCR) kit (Takara, Otsu, Japan). Primers were designed according to Pubmed Genbank and synthesized by Shanghai Sangon Biological Engineering and Technology and Service Co. Ltd (China) (Table 1). Briefly, PCR was performed at 95°C with initial denaturation for 15 secs, followed by 45 cycles of amplification at 95°C (5 secs) and 60°C (30 secs). Finally, the samples were extended at 72°C for 10 min. For each gene, crossing point (Cp) values were determined and normalized by subtraction of the Cp value for GAPDH (generating a ΔCp value). Relative change was determined by subtraction of the ΔCp value for the control sample from the ΔCp value for the treated sample (ΔΔCp value). Fold change was subsequently calculated using the formula 2^−ΔΔCp^, which was denoted as the relative mRNA expression of each gene. Aliquots of the synthesized PCR products were separated by electrophoresis on a 1.5% agarose gel and analyzed by Gel-Pro 3.1 software.

**Table 1:** PCR primer sequences for RT-PCR detection.

GAPDH	114 bp	ForwardReverse	5′-TTCAACGGCACAGTCAAGG-3′ 5′-CTCAGCACCAGCATCACC-3′
Cnr1	142 bp	ForwardReverse	5′-GACCTACCTGATGTTCTGGATTG-3′ 5′-GTGGATGATGATGCTCTTCTGG-3′
α-SMA	191 bp	ForwardReverse	5′-CACCATCGGGAATGAACG-3′5′-TGTCAGCAATGCCTGGGTA-3
PDGFRβ	233 bp	ForwardReverse	5′-GCCATCCTGAGGTCCCAA-3′ 5′-TCGCAGGAGATGGTGGAAG-3′
TGFβ1	301 bp	ForwardReverse	5′-GCAACAACGCAATCTATGAC-3′ 5′-TTCCTCTGCCTTATGTCCC-3′
TGFβR II	193 bp	ForwardReverse	5′-TCACCTACCACGGCTTCAC-3′ 5′-ACGCCCGTCACTTGGATA-3′
Fibronectin	312 bp	ForwardReverse	5′-TCAGCTGTACCATTGCAAATC-35′-TGGTGTCCTGATCATTGCAT-3′
Procollagen Type I	142 bp	ForwardReverse	5′-CCTCCCAGCGGTGGTTAT-3′ 5′-GGCTCTTGAGGGTAGTGTCC-3′
E-cadherin	196 bp	ForwardReverse	5′-GTCAAACGGCATCTAAAGC-3′ 5′-TCAGACCCTGGTGAAAGC-3′
Snail	216 bp	ForwardReverse	5′-TCCTTGCTCCACAAACACCA-3′ 5′-TGCCTTCCATCAGCCATCT-3′
Vimentin	132 bp	ForwardReverse	5′-TCCCTGAACCTGAGAGAAAC-3′ 5′-ATCGTGGTGCTGAGAAGTC-3′

### Proliferation assay

After lentivirus transfected and/or PDGF-BB stimulation, primary HSCs cultured in 96 wells plate were stimulated with 10 µl/well 5-bromodeoxyuridine (BrdU, Roche, Mannheim, Germany) for 24 h, followed by being fixed with 200 µl/well FixDenat solution for 30 minutes. Cells were then incubated with 5–10% BSA for 30 minutes. Subsequently, 100 µl/well anti-BrdU-POD solutions were added to the cells. The immune complexes are detected by the subsequent substrate reaction. The reaction product is quantified by measuring the absorbance at the 450 nm wavelength using a scanning multiwell spectrophotometer (ELISA reader). The developed color and thereby the absorbance values directly correlate to the amount of DNA synthesis hereby to the number of proliferating cells in the respective microcultures. Results were expressed as mean±SD of cells that had incorporated BrdU (percentage of BrdU-positive cells).

### Apoptosis assay

Apoptosis assay was performed according to the protocol of the Annexin V/PI Apoptosis Assay Kit (Jingmei, Shanghai, China). In brief, primary HSCs stimulated by AM251 and/or 2-AG and/or transfected by lentivirus mentioned above were harvested and washed in ice-cold phosphate-buffered saline. The cells were then resuspended in 250 µl binding buffer to a final concentration of 1×10^6^ cells per ml, and 100 µl of the cell suspension was aliquot into each test tube. Five µl of Annexin/FITC and 10 µl PI (20 µg/ml) were added. FACS Aria Flow Cytometer System (BD, San Jose, USA) was used to analyze cell apoptosis.

### Western blot analysis

After lentivirus transfection and/or AM251 treatment, whole cells were homogenized in lysis buffer. Phosphatase inhibitor (Sigma, St Louis, USA) was added to prevent protein dephosphorylation. A Bio-Rad Rapid Coomassie kit (Bio-Rad, Hercules, CA) was used to determine the total protein concentration. Sixty micrograms of protein were run on a 10% SDS-poly-acrylamide gel and transferred to a polyvinylidene difluoride membrane. Immunoblotting was performed with primary antibodies against CB1 (Alomone labs, CA, USA), αSMA (Boster, Wuhan, China), TGF-β1 (CST, Beverly, USA), transforming growth factor β receptor II (TGF-β RII, CST, Beverly, USA), fibronectin (Santa Cruz, Santa Cruz, USA), procollagen Type I (Santa Cruz, Santa Cruz, USA), E-cadherin (Santa Cruz, Santa Cruz, USA), snail (CST, Beverly, USA), vimentin (CST, Beverly, USA) and GAPDH (Santa Cruz, Santa Cruz, USA), followed by incubation with horseradish peroxidase-conjugated anti-mouse- or anti-rabbit IgG (Santa Cruz, Santa Cruz, CA, USA) as secondary antibodies. The blot was developed with a chemiluminescence system (ECL; Amersham, Piscataway, NJ) according to the manufacturer's protocol. The optical densities of the bands were measured with a Model GS-700 Imaging Densitometer (Bio-Rad, Hercules, CA).

### Animal model of hepatic fibrosis

All animals (male SD rats) weighing about 160 g were housed in cages with stainless steel wire tops and with 12-h light-dark cycles under standard animal laboratory conditions in the SPF-grade animal room of the experimental animal center of General Hospital of PLA in China. The rats had free access to standard rat chow and water. This study was carried out in strict accordance with the recommendations in the Guide for the Care and Use of Laboratory Animals of the National Institutes of Health. The protocol was approved by the Local Ethical Committee of General Hospital of PLA (Permit Number: 2012-87).

To induce the hepatic fibrosis animal model, 48 male SD rats were randomly divided into the normal group (n = 12) and the DMN (dimethylnitrosamine, Tianjin Chemical Reagent Research Institute, Tianjin, China)-treatment group (n = 36). All the animals in the DMN-treatment group received DMN dissolved in saline at a dose of 10 ml DMN/kg by a peritoneal injection for three consecutive days each week for 3 weeks, and were randomly assigned to the following three groups (12 in each): CB1-RNAi-LV treatment group (1×10^8^ TU CB1-RNAi-LV dissolved in Ringer's solution was injected through the tail vein on the day before DMN injection), Ringer's solution group and NC-LV treatment group (1×10^8^ TU NC-LV dissolved in Ringer's solution was injected through the tail vein on the day before DMN injection) as the negative control group.

At the end of 3 weeks, the rats were killed and serum samples were collected for biochemical tests. The right lobes of livers were taken and soaked with 10% neutral formaldehyde for histology.

### Evaluation of hepatic fibrosis

To evaluate the pathogenesis of hepatic fibrosis semiquantitatively, Masson's trichrome staining was used. The stage of fibrosis was graded as follows: stage 0, normal connective tissue (no fibrosis); stage 1, fibrous portal expansion; stage 2, periportal fibrosis with short septa extending into lobules or rare porto-portal septa (intact architecture); stage 3, fibrous septa reaching adjacent portal tracts and terminal hepatic venule (architecture distortion, but no obvious cirrhosis); stage 4, diffuse nodular formation (cirrhosis).

### Immunohistochemistry examination for liver sections

The paraffin sections were deparaffinized using xylene and alcohol and hydrated to water, then treated with primary antibodies including α-SMA polyclone antibody, CB1 polyclone antibody and E-cadherin monoclone antibody, followed by biotinylated second antibody and then streptavidin peroxidase. The integrated optical intensity of α-SMA, CB1 and E-cadherin was semiquantified using Image-Pro Plus software.

### Statistical analysis

Results were presented as means of three independent experiments (±SD). In the semiquantitive analysis of histological staging, nonparametric tests (Wilcoxon test) were used and other statistical analyses performed with an unpaired Student's t-test. Differences were considered as significant or highly significant at *p*<0.05 or *p*<0.01, respectively.

## Results

### RNA interference knockdown the expression of CB1 in HSCs

**Figure 1:**
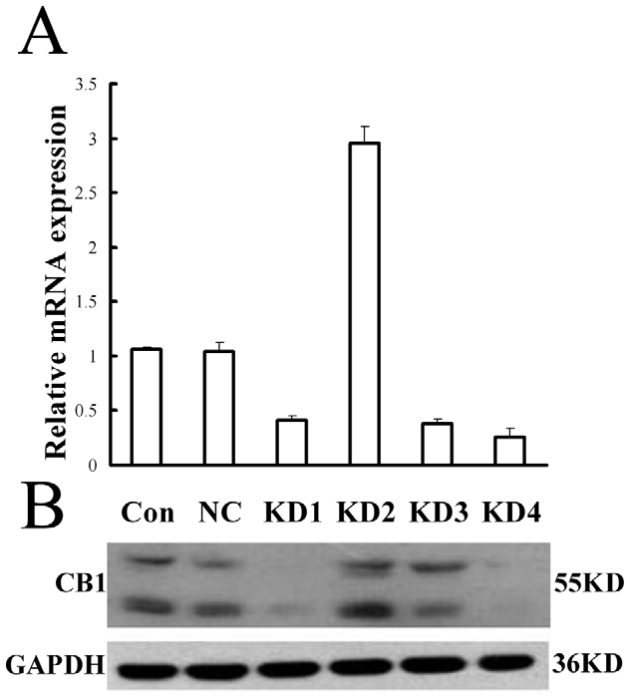
Effect of shRNA expressing lentivirus against CB1 on CB1 expression in primary hepatic stellate cells. (**A**) The statistical results of CB1 mRNA expression in primary hepatic stellate cells (HSCs) transfected with 4 pairs of shRNA expressing lentivirus named KD1, KD2, KD3 and KD4 by RT-PCR detection. The pGCSIL/U6 mock vector named Con was used as a negative control. KD4 displayed the highest gene-silencing efficacy among all the 4 pairs of lentivirus. Relative expression levels of mRNA were normalized against those of GAPDH mRNA. (**B**) The CB1 protein expression in HSCs transfected with 4 pairs of shRNA expressing lentivirus and the negative control lentivirus, respectively. The results demonstrated that KD4 deduced CB1 mRNA expression by about 75%, and protein expression by about 80%, which had the highest gene-silencing efficacy among all the four pairs of lentivirus vectors.

In this study, we constructed four pairs of shRNA expressing lentivirus vectors named KD1, KD2, KD3 and KD4 to knockdown CB1 mRNA expression in the activated HSCs. RT–PCR detection showed that KD4 had the highest gene-silencing efficacy among all the four pairs of lentivirus vectors (Figure 1A). KD4 deduced CB1 mRNA expression by about 75% at 24 h, and protein expression by about 80% at 48 h, respectively (Figure 1B). So in the subsequent experiments, we selected KD4 (also named CB1-RNAi-LV in the ensuing paragraphs) to knock down CB1 expression and investigate its effect on hepatic fibrosis in vitro and in vivo.

### CB1-RNAi-LV inhibits the activation and ECM production of HSCs

**Figure 2:**
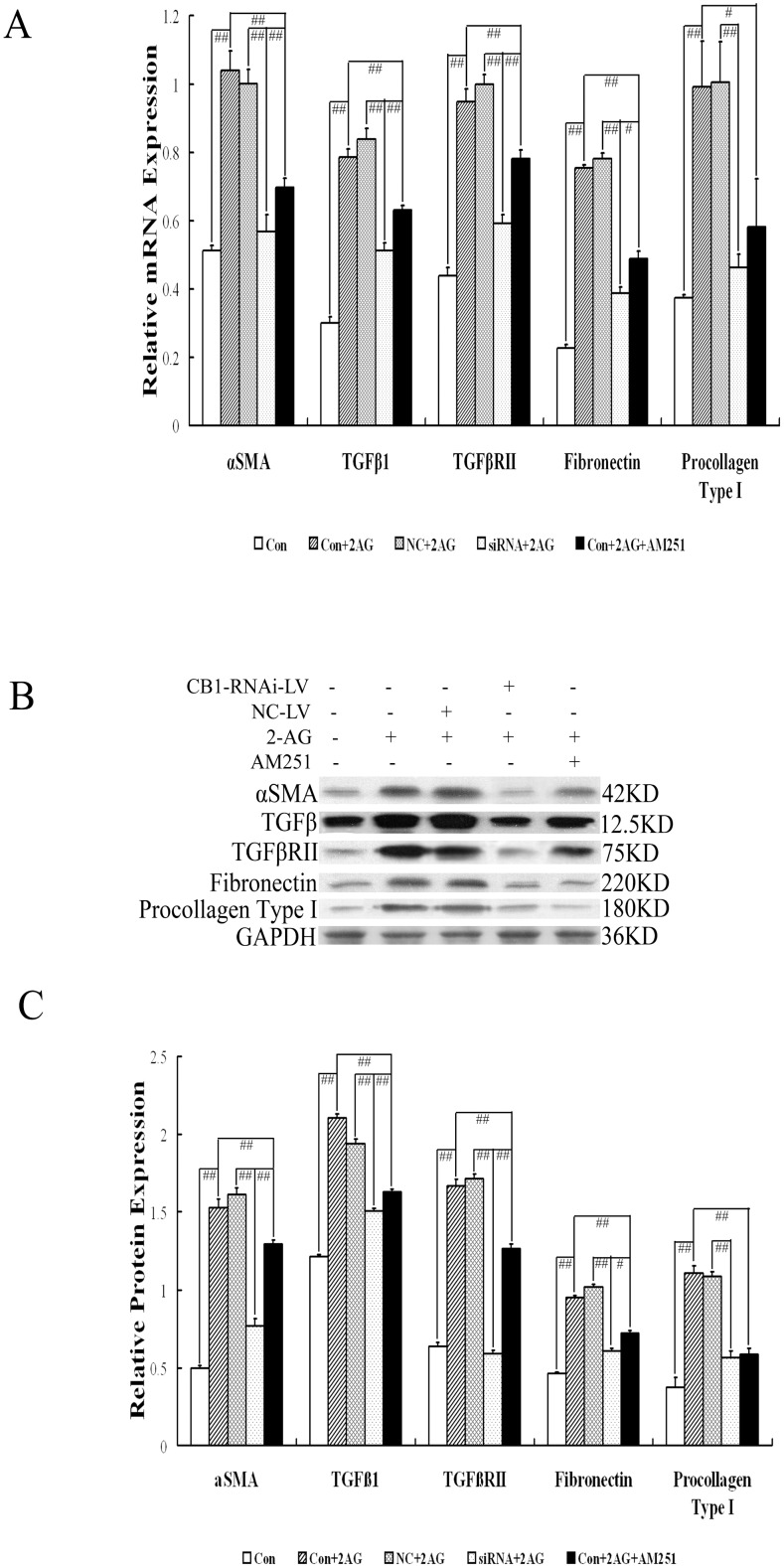
Effect of CB1-RNAi-LV on activation and ECM production of primary hepatic stellate cells. (**A**) The statistical results of α-SMA, TGF-β1, TGF-β RII, fibronectin and procollagen type I mRNA expression in HSCs (named Con), HSCs with 2-AG (named Con+2AG), HSCs with NC-LV and 2-AG (named NC+2AG), HSCs with CB1-RNAi-LV and 2-AG (named siRNA+2AG), HSCs with 2-AG and AM251 (named Con+2AG+AM251) by RT-PCR analysis. Relative expression levels of mRNA were normalized against those of GAPDH mRNA. (**B**) shows representative graphs of α-SMA, TGF-β1, TGF-β RII, fibronectin and procollagen type I protein expression by Western blot analysis and (**C**) shows the statistical results. Results demonstrated that the mRNA and protein expressions of α-SMA, TGF-β1, TGF-β RII, fibronectin and procollagen type I in HSCs were all increased significantly after 2-AG stimulation, which were all decreased significantly when stimulated with AM251 or transfected with CB1-RNAi-LV. Furthermore, the mRNA expression of α-SMA, TGF-β1, TGF-β RII and fibronectin in HSCs stimulated by 2-AG and AM251 was higher significantly than that in HSCs stimulated by 2-AG and transfected with CB1-RNAi-LV. “#” *p*<0.05, “# #” *p*<0.01.

To investigate the effect of CB1-RNAi-LV on the activation and ECM production of HSCs, we detected mRNA and protein expression of α-SMA, TGF-β1, TGF-β RII, fibronectin and procollagen type I by RT-PCR and western blot. The results showed that the expressions of all the five molecules in HSCs increased significantly by 2-AG stimulation or by 2-AG stimulation and NC-LV transfection, and the expressions of all in HSCs with 2-AG stimulation decreased significantly when stimulated by AM-251, the CB1 antagonist (Figure 2). In HSCs stimulated by 2-AG and transfected with CB1-RNAi-LV, mRNA and protein expression of the five molecules also decreased markedly, compared with that in HSCs stimulated by 2-AG and transfected with NC-LV (Figure 2). Furthermore, mRNA and protein expression of α-SMA, TGF-β1, TGF-β RII and fibronectin in HSCs with 2-AG stimulation and CB1-RNAi-LV transfection declined significantly, when compared with that in HSCs stimulated by 2-AG and treated with AM251. We deduced that CB1 could promote the activation and ECM production of HSCs, which could be blocked by CB1 siRNA and CB1 antagonist, and the inhibition effect of CB1 siRNA on HSCs activation was more significant than the CB1 antagonist.

### CB1-RNAi-LV suppresses HSC proliferation induced by PDGF-BB

**Figure 3:**
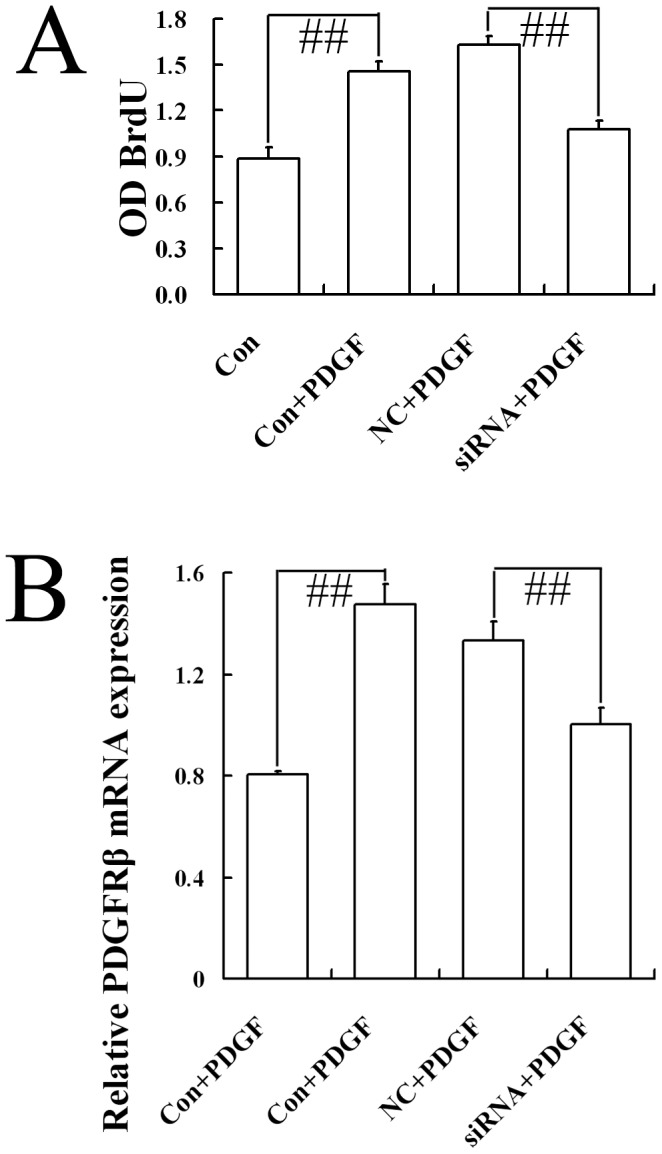
Effect of CB1-RNAi-LV on the proliferation of primary hepatic stellate cells. (**A**) The statistical results of the optical density (OD) value of BrdU incorporative cells in HSCs (named Con), HSCs with PDGF (named Con+PDGF), HSCs with NC-LV and PDGF (named NC+PDGF), HSCs with CB1-RNAi-LV and PDGF (named siRNA+PDGF) by ELISA method. “# #” *p*<0.01. Results showed that the proliferation of HSCs stimulated by PDGF-BB elevated significantly than HSCs alone. NC-LV transfection did not affect the proliferation of HSCs stimulated by PDGF-BB. Furthermore, the proliferation of HSCs stimulated with PDGF-BB and transfected with CB1-RNAi-LV decreased markedly than that of HSCs stimulated with PDGF-BB and transfected with NC-LV. (**B**) The statistical results of PDGFR-β mRNA expression in HSCs (named Con), HSCs with PDGF (named Con+PDGF), HSCs with NC-LV and PDGF (named NC+PDGF), HSCs with CB1-RNAi-LV and PDGF (named siRNA+PDGF) by RT-PCR analysis. Relative expression levels of mRNA were normalized against those of GAPDH mRNA. “# #” *p*<0.01. Results showed that PDGFR-β mRNA expression increased markedly by PDGF-BB pretreatment or by PDGF-BB pretreatment plus NC-LV transfection, while PDGFR-β mRNA expression in HSCs pretreated with PDGF-BB and transfected with CB1-RNAi-LV decreased markedly than that in HSCs pretreated with PDGF-BB and transfected with NC-LV.

PDGF is recognized as the most potent mitogen for HSCs, which impedes HSCs proliferation via its receptor β subunit (PDGFR-β). To investigate the effect of CB1-RNAi-LV on HSCs proliferation pretreated with PDGF-BB, BrdU incorporation was used. Results showed that PDGF-BB significantly promoted HSCs proliferation, which was constrained by CB1-RNAi-LV (Figure 3A). Furthermore, RT-PCR detection showed that PDGFR-β mRNA expression in HSCs increased markedly by PDGF-BB pretreatment, and decreased markedly by CB1-RNAi-LV transfection (Figure 3B). It was deduced that CB1 siRNA could suppress PDGFR-β mRNA expression, and inhibit HSCs proliferation induced by PDGF.

### CB1-RNAi-LV does not affect HSCs apoptosis induced by 2-AG

It is recognized that hepatic fibrosis is reversible by apoptosis of hepatic MFs [1]. In this study, results showed that the percentage of apoptotic HSCs increased markedly by 2-AG stimulation (14.3±3.8% vs 7.4±2.1%, *p*<0.01), and no significant differences of the percentage of apoptotic HSCs were shown between HSCs with 2-AG stimulation and HSCs with 2-AG stimulation and NC-LV transfection (14.3±3.8% vs 12.9±4.3%, *p*>0.05). Furthermore, there were also no significant differences of the percentage of apoptotic HSCs between HSCs with 2-AG stimulation and NC-LV transfection and HSCs with 2-AG stimulation and CB1-RNAi-LV transfection (12.9±4.3% vs 14.1±5.3%, *p*>0.05), and between HSCs with 2-AG stimulation and HSCs with 2-AG stimulation and AM251 treatment (14.3±3.8% vs 13.6±4.7%, *p*>0.05). These data revealed that 2-AG could increase the apoptosis of culture-activated HSCs, but neither CB1 shRNA nor CB1 antagonist AM251 could affect the HSCs apoptosis induced by 2-AG, indicating that 2-AG-induced HSCs apoptosis was independent of its receptor CB1.

### CB1-RNAi-LV induce Mesenchymal-Epithelial Transition in HSCs

**Figure 4:**
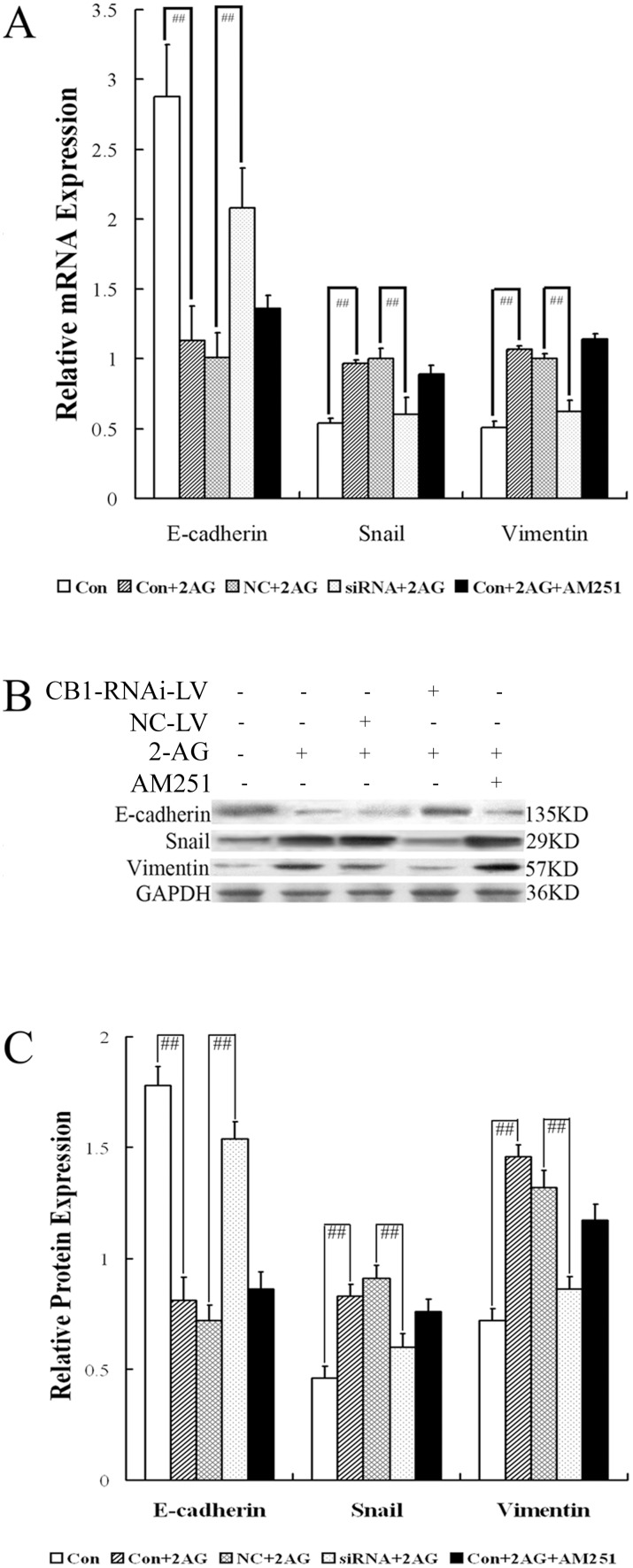
Effect of CB1-RNAi-LV on Mesenchymal-Epithelial Transitions of primary hepatic stellate cells. (**A**) The statistical results of E-cadherin, Snail and Vimentin mRNA expression in HSCs (named Con), HSCs with 2-AG (named Con+2AG), HSCs with NC-LV and 2-AG (named NC+2AG), HSCs with CB1-RNAi-LV and 2-AG (named siRNA+2AG), HSCs with 2-AG and AM251 (named Con+2AG+AM251) by RT-PCR analysis. Relative expression levels of mRNA were normalized against those of GAPDH mRNA. “# #” *p*<0.01. (**B**) shows representative graphs of E-cadherin, Snail and Vimentin protein expression by Western blot analysis and (**C**) shows the statistical results. These data indicated that the mRNA and protein expressions of E-cadherin in HSCs were down-regulated significantly by 2-AG stimulation, while the levels of Snail and Vimentin were up-regulated significantly by 2-AG stimulation. On the other hand, the expression level of E-cadherin in HSCs with CB1-RNAi-LV transfection and 2-AG stimulation were increased markedly than that in HSCs with NC-LV transfection and 2-AG stimulation, while the expression levels of Vimentin and Snail in HSCs with CB1-RNAi-LV transfection and 2-AG stimulation were decreased markedly than that in HSCs with NC-LV transfection and 2-AG stimulation. The results also revealed that the expression levels of E-cadherin, Snail and Vimentin in HSCs stimulated by 2-AG were not affected by AM251 treatment.

Several lines of evidence support an important role of Epithelial-to-Mesenchymal Transition (EMT) and Mesenchymal-to-Epithelial Transition (MET) in the pathogenesis of hepatic fibrosis [15], [16]. RT-PCR and western blot analysis indicated that the levels of E-cadherin, the epithelial phenotype gene, were down-regulated significantly in the HSCs stimulated by 2-AG, compared with the HSCs alone (Figure 4), and were up-regulated significantly in the HSCs transfected by CB1-RNAi-LV, compared with the HSCs transfected by NC-LV (Figure 4). These data indicated that the CB1 siRNA could promote the epithelial phenotype expression in activated HSCs. On the other hand, the expression of vimentin and snail in HSCs, which indicated mesenchymal phenotype and mesenchymal defining function, was significantly up-regulated by 2-AG stimulation, which could be down-regulated by CB1-RNAi-LV transfection (Figure 4). These data demonstrated that deactivation of activated HSCs could be achieved by inducing the MET process ascribed to down-regulation of CB1, revealing an important role of CB1 for HSCs in restoring biological characteristics of activated state.

### CB1-RNAi-LV relieves liver injury in DMN rats

**Figure 5:**
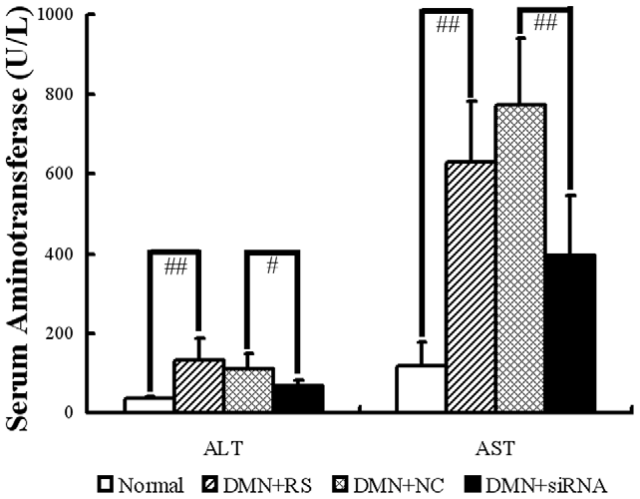
Effect of CB1-RNAi-LV on serum aminotransferase in DMN induced hepatic fibrosis. The statistical results of serum alanine aminotransferase (ALT) and aspartate aminotransferase (AST) levels in normal rats, rats with DMN treatment and Ringer's solution (RS) injected, rats with DMN treatment and NC-LV injected, rats with DMN treatment and CB1-RNAi-LV injected. “#” *p*<0.05 and “# #” *p*<0.01. The data demonstrated that serum ALT and AST elevated significantly in DMN rats as compared with the normal rats. However, CB1-RNAi-LV treatment markedly reduced the serum ALT and AST as compared with NC-LV treatment rats.

To further investigate the effect of CB1-RNAi-LV on liver injury, the DMN rats model was used. Serum levels of alanine aminotransferase (ALT) and aspartate aminotransferase (AST) elevated significantly in DMN rats as compared with the normal rats (Figure 5). However, CB1-RNAi-LV treatment markedly reduced the serum levels of ALT and AST as compared with NC-LV treatment (Figure 5), illustrating that CB1 siRNA could relieve liver injury induced by DMN.

### CB1-RNAi-LV knocked down CB1 expression in DMN rats

**Figure 6:**
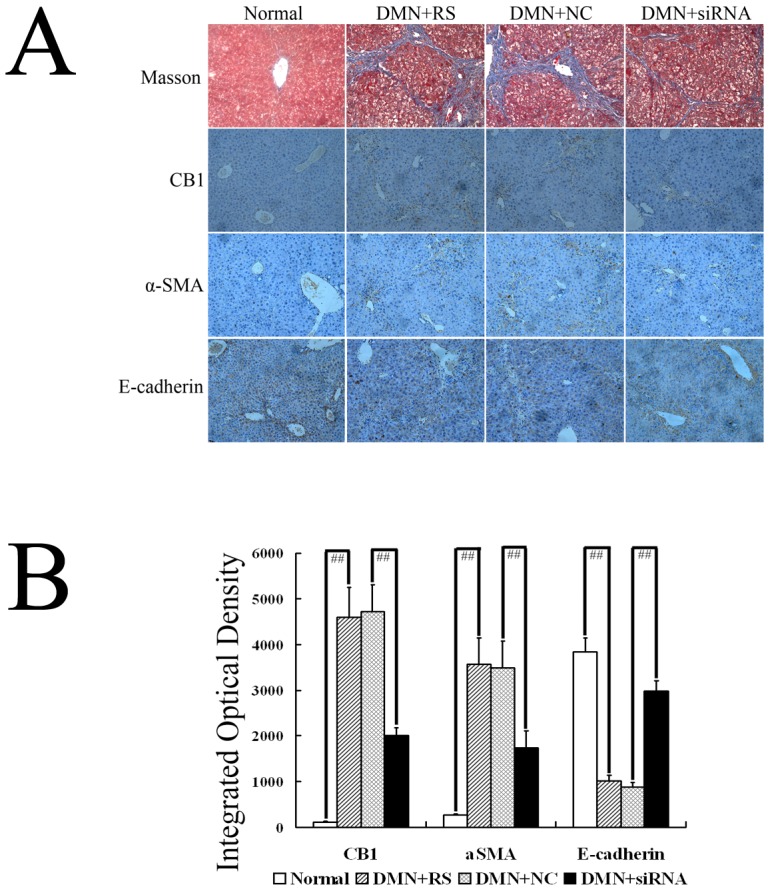
Effect of CB1-RNAi-LV on hepatic fibrosis and epithelial- mesenchymal transitions in livers of DMN rats. (**A**) The first line showed the representative graph of Masson's trichrome staining (magnification of 20×) in normal rats (Normal), rats with DMN treatment and Ringer's solution injected (DMN+RS), rats with DMN treatment and NC-LV injected (DMN+NC-LV), rats with DMN treatment and CB1-RNAi-LV injected (DMN+RNAi-LV). The second, the third and the fourth line showed the representative graph of CB1, α-SMA and E-cadherin immunohistochemical examination (magnification of 20×) in normal rats (Normal), rats with DMN treatment and Ringer's solution injected (DMN+RS), rats with DMN treatment and NC-LV injected (DMN+NC-LV), rats with DMN treatment and CB1-RNAi-LV injected (DMN+RNAi-LV). (**B**) Shows statistical results of CB1, α-SMA and E-cadherin immunohistochemical staining. Masson's trichrome staining of liver sections in the DMN rats and the NC-LV treatment rats showed periportal fibrosis with short septa extending into lobules or porto-portal septa, and severe cholestasis and bile duct hyperplasia were also observed, whereas hepatic fibrosis was significantly ameliorated in the CB1-RNAi-LV treatment rats as compared with the NC-LV treatment rats. Immunohistochemical examination revealed that CB1 and α-SMA protein expressions increased significantly in the DMN rats, which were suppressed by CB1-RNAi-LV treatment. Immunochemical examination also showed that DMN treatment declined E-cadherin expression significantly compared with normal rats, while E-cadherin expression elevated significantly in DMN rats transfected with CB1-RNAi-LV, compared with that in DMN rats transfected with NC-LV. “# #” *p*<0.01.

We have confirmed that CB1-RNAi-LV could suppress CB1 expression in primary isolated HSCs, so we transfected DMN rats with CB1-RNAi-LV to identify the efficacy of CB1-RNAi-LV on CB1 expression in vivo. Immunohistochemical examination showed almost no CB1 expression in normal liver, which increased markedly in DMN rats. However, CB1-RNAi-LV transfection markedly decreased the expression of CB1 in DMN rats, revealing that CB1 siRNA could also knock down CB1 expression in vivo (Figure 6).

### CB1-RNAi-LV inhibits HSC activation and ameliorates hepatic fibrosis in DMN rats

Immunohistochemical examination revealed that CB1 and α-SMA protein expressions increased significantly in the DMN rats, which were suppressed by CB1-RNAi-LV treatment (Figure 6). Masson's trichrome staining of liver sections in the DMN rats and the NC-LV treatment rats showed periportal fibrosis with short septa extending into lobules or porto-portal septa, and severe cholestasis and bile duct hyperplasia were also observed, the semiquantitative analysis of the fibrosis stage pathologically showed that hepatic fibrosis was significantly ameliorated in the CB1-RNAi-LV treatment rats as compared with the NC-LV treatment rats (Figure 6A and Table 2). These results clearly indicated that CB1 shRNA could ameliorate hepatic fibrosis induced by DMN.

**Table 2:** Effect of CB1 shRNA on hepatic fibrosis of DMN rats.

Group/Stage	0–1	1–2	2–3	3–4	U
Normal	12	0	0	0	/
DMN+RS	0	0	10	0	4.3875**
DMN+NC-LV	0	0	6	2	/
DMN+RNAi-LV	0	8	0	0	3.4668##

To score the stage of fibrosis, three fields of microscope in the liver section of each rat were scored, and the average score was the stage of fibrosis.

** *p*<0.01 compared with the normal rats.

## *p*<0.01 compared with the DMN+NC-LV rats.

### CB1-RNAi-LV induces Mesenchymal-Epithelial Transition in livers of DMN rats

To further investigate whether CB1-RNAi-LV could induce the MET process in hepatic fibrosis animal model, we treated hepatic fibrosis rats induced by DMN with CB1-RNAi-LV or its negative control, NC-LV. Immunochemical examination showed that DMN treatment declined E-cadherin expression significantly compared with normal rats, while E-cadherin expression elevated significantly by CB1-RNAi-LV transfection, compared with NC-LV transfected rats (Figure 6). These results demonstrated that EMT played important roles in the progression of hepatic fibrosis, which can be reversed by CB1 siRNA.

## Discussion

Many studies have demonstrated the up-regulation of the expression of CB1 in hepatic MFs and vascular endothelial cells, as well as increased concentration of endocannabinoids in liver in the course of chronic progressive liver diseases [8], [9], [13], [17]. Teixeira-Clerc et al [9] have provided evidence for the involvement of CB1 in regulation of hepatic fibrosis and the profibrogenic effect of CB1 signaling. Moreover, the effect of CB1 inactivation was demonstrated in three experimental models of liver injury induced by CCl4, thioacetamide or biliary cholestasis. The favorable antifibrogenic results were obtained either by pharmacological inactivation with rimonabant, a selective antagonist of CB1 receptor, or via genetic inactivation in homozygous CB1-deficient mice, and decreased progression of fibrosis was accompanied by reduced hepatic TGF-β1 expression, and inhibited proliferation and increased apoptosis of MFs. In the present study, RNA interference technique was used firstly to knockdown the CB1 expression, and lentivirus based shRNA expressing vector named CB1-RNAi-LV was applied firstly in hepatic fibrosis animal model.

Several lines of evidence indicate that TGF-β1 from autocrine or paracrine sources plays a role in activating HSCs and increasing the synthesis of ECM proteins and cellular receptors for various matrix proteins [18], [19]. In response to TGF-β1, type II TGF-β1 receptors autophosphorylated and transmitted the signal by which regulatory Smad molecules (Smad3/2) are phosphorylated and form an active complex with co-Smad (Smad4) [20]. The fibrogenic effect of TGF-β in liver can be inhibited by neutralizing antibodies against TGF-β or soluble TGF-β-RII [21], [22]. In the present study, reduced expressions of TGF-β1, its receptor TGF-β RII and snail, the important intracellular transcription factor closely related to the intracellular signaling transduction pathway of TGF-β1, were induced by CB1-RNAi-LV in cultured HSCs, furthermore, the antifibrogenic effect of CB1-RNAi-LV was also confirmed in vitro and in vivo, demonstrating that the restorative effect of CB1-RNAi-LV on hepatic fibrosis might be partially attributed to the decreased expression of TGF-β1 and its receptor, and the blockage of its intracellular signaling transduction pathway subsequently.

Epithelial cells are adherent cells that closely attach to each other, forming coherent layers in which cells exhibit apico-basal polarity. Mesenchymal cells, in contrast, are non-polarized cells, capable of moving as individual cells because they lack intercellular connections. EMT describes the process by which cells gradually lose their epithelial signatures while acquiring the characteristics of mesenchymal cells. MET refers to the reverse process. Organ fibrosis is a potential outcome of EMT, which is associated with inflammation, when inflammation persists, EMT generates fibroblastic cells that accumulate and cause progressive fibrosis [23].

A series of recent studies strongly suggested that EMT is an important contributor to the progression of hepatic fibrosis [16], [24]–[26]. The adhesion junction protein E-cadherin is critical for the disappearance of cell-cell junctions and apical-basal polarity of epithelia. As one of intermediate filament proteins, vimentin will accumulate dramatically in the cytoplasm during EMT. The expression of E-cadherin can be repressed by transcriptional factors including snail [27], [28]. HSCs are the most accepted myofibroblast progenitors in the liver [19]. Recently, several studies pointed out that HSCs in the quiescent state contain epithelial characteristics, and the activation of HSCs might be considered as an EMT phenomenon [25], [29], [30].

The paracrine activity of HSCs may also affect the cell phenotype of hepatocytes, the main epithelial cells in liver, which may be another mechanism of EMT in liver. Jeong WI, et al [31] reported chronic alcohol feeding could promote CB1 expression in hepatocytes in vivo, which could be replicated in vitro by co-culturing control hepatocytes with HSCs isolated from ethanol-fed mice, implicating HSCs-derived mediators in the regulation of hepatic CB1. HSCs are a rich source of retinoic acid (RA). Mukhopadhyay B et al [10] confirmed that the HSCs-derived 2-AG also promoted CB1 expression in hepatocytes, which was mediated indirectly via RA, because it was absent in hepatocytes from mice lacking retinaldehyde dehydrogenase1, the enzyme catalyzing the generation of RA from retinaldehyde.

Since EMT is dynamic and bidirectional, we deduce that fibrogenic cells such as activated HSCs can undergo MET and revert back to an epithelial phenotype, which results in the reverse of hepatic fibrosis. Actually, bone morphogenic protein-7, a negative regulator of EMT, has been proven as an effective agent to control liver fibrosis [32].

In our present study, we have confirmed that expression of vimentin, snail and α-SMA was increased during HSCs activation, accompanying the down-regulation of E-cadherin. Significant different expression of both epithelial and mesenchymal cell markers strongly suggests that HSCs activation is closely related to the EMT process. More importantly, our study has clearly demonstrated that decreased expression of CB1 gene could increase the expression of epithelial markers in favor of down-regulating mesenchymal markers expression and suppressing the known fibroblast functions. In addition, we also demonstrated that CB1 siRNA deactivates the MFs through inducing the MET process of activated HSCs and robustly inhibited their proliferation. All of these results provided direct evidence for the potential role of EMT in activation of HSCs and suggested that CB1 siRNA could block and reverse liver fibrosis partly through the inhibition of the EMT state of activated HSCs.

It was reported that TGF-β1 was important triggers to EMT in fibrotic liver [18]. During EMT, TGF-β1 inhibits E-cadherin expression by up-regulating transcriptional repressors such as Snail, Zeb, and Twist [27], [28]. This process is receptor-dependent. Considering the downregulation of TGF-β1 and its receptor TGF-β RII, and inhibition of EMT in HSCs transfected by CB1-RNAi-LV, we deduced that the decrease expression of CB1 could promote MET in HSCs via suppressing the expression of TGF-β1 and its intracellular signal transduction subsequently.

E-cadherin is an epithelial marker. Il Je Cho et al [33] reported that overexpression of E-cadherin could reduce the expression of TGF-β and block the intracellular Smad signaling pathway mediated by TGF-β in HSCs. We provide the hypothesis that a negative feedback exists in Smad signaling pathway mediated by TGF-β and the E-cadherin expression: TGF-β promotes EMT of HSCs via Smad signaling pathway and results in decreased expression of E-cadherin subsequently, but overexpression of E-cadherin also inhibit Smad signaling pathway and decrease the expression of TGF-β.

As one major endocannabinoids ligands, 2-AG is the likely fibrogenic mediator because its hepatic level is preferentially increased in the CCl4 treated mice [3], [4]. Previous study reported that 2-AG induce apoptosis in activated HSCs in vitro, which means that 2-AG has antifibrotic activity in vitro [34], [35]. In the present study, we also found that extrinsic supplement of 2-AG could incresse HSCs apoptosis, but 2-AG-induced HSCs apoptosis was independent of its receptor CB1, which was in accordance with the previous study [34], [35]. It means that the ameliorate effect on hepatic fibrosis by CB1 shRNA can not explained by the increase of HSCs apoptosis.

AM251, the CB1 antagonists, was added to the activated HSCs to inactivate CB1 receptor in the present study. Results showed decreased expression of α-SMA, TGF-β1, TGF-β RII, fibronectin and procollagen Type I, and reduced ECM production after AM-251 treatment in HSCs, indicating the antifibrotic effect of AM251 on hepatic fibrosis in vitro, which was agreed with the previous study reported by Yang YY et al [36], in which AM-251 treatment could inhibit hepatic TGF-β1 expression and reduce hepatic collagen deposition in cirrhotic livers induced by common bile-duct ligation. Interestingly, our study also revealed that the antifibrotic effect of CB1 siRNA on hepatic fibrosis was superior to AM251. These data indicated that gene therapy based on siRNA had superiority over chemical therapy based on receptor antagonists.

In summary, the present study first provides strong evidence for the striking suppression effect of CB1 siRNA on hepatic fibrosis, which is associated with inhibition of activation and proliferation of HSCs. This study also confirmed firstly that the suppression effect of CB1 siRNA on hepatic fibrosis was superior to CB1 antagonist. Furthermore, reversal of EMT of HSCs secondary to CB1 knocking down is also defined firstly in this study. These results suggest that CB1 siRNA might emerge as a therapeutic agent to control liver fibrosis. In this study, we also present the MET of HSCs in the fibrotic liver as a new therapeutic strategy for chronic liver diseases.
